# Removal of Gutta-Percha/Zinc-Oxide-Eugenol Sealer or Gutta-Percha/Epoxy Resin Sealer from Severely Curved Canals: An In Vitro Study

**DOI:** 10.1155/2011/541831

**Published:** 2011-11-15

**Authors:** Santhoshini Reddy, Prasanna Neelakantan, Mohammad Ali Saghiri, Mehrdad Lotfi, Chandragiri Venkata Subbarao, Franklin Garcia-Godoy, James L. Gutmann

**Affiliations:** ^1^Department of Conservative Dentistry and Endodontics, Saveetha Dental College and Hospitals, Saveetha University, Chennai 600 077, India; ^2^Department of Dental Material, Islamic Azad University, Dental Branch, Tehran, Iran; ^3^Research Center for Pharmaceutical Nanotechnology and Department of Endodontics, Dental Faculty, Tabriz University (Medical Sciences), Tabriz, Iran; ^4^Bioscience Research Center, College of Dentistry, The University of Tennessee Health Science Center, Memphis, TN, USA; ^5^Baylor College of Dentistry, Texas A&M Health Science Center, Dallas, Texas, USA

## Abstract

The aim of this study was to compare the cleanliness of root canal walls after retreatment using two rotary retreatment files to hand instruments in severely curved canals obturated with gutta-percha and two different sealers. Single rooted mandibular premolars (*n* = 90) with root curvatures were instrumented and obturated with gutta-percha and an epoxy resin (Group 1, *n* = 45) or zinc oxide eugenol sealer (Group 2, *n* = 45). Following retreatment of the specimens (*n* = 15 ProTaper Universal Retreatment Files (Subgroup B) or R-Endo retreatment files (Subgroup C) after 1 month, split specimens were examined under a stereomicroscope and the percentage of remaining root filling material was statistically compared using one way ANOVA with Bonferroni adjustment for multiple comparisons (*P* = 0.05). The R-Endo system performed significantly better than the other two file systems (*P* < 0.05). None of the systems used in this study cleaned root canals thoroughly. The R-Endo system did provide cleaner walls when compared to the other instruments used. The type of root filling materials had an impact on the outcomes with all techniques.

## 1. Introduction

Necrotic tissues, bacteria, and biofilms have been identified, along with coronal leakage, vis-a-vis poor restorations, recurrent caries, tooth fractures, or extensive periodontal disease, as the causes for persistent periapical disease following root canal treatment [1]. Elimination of these etiologies is essential to reestablish an environment conducive to repairing and healing. This implies that if nonsurgical revision is the treatment of choice then not only must these etiologic factors be removed, but also the filling material present in the root canal system must be eliminated [2, 3]. The literature is replete with studies that have discussed the techniques for removal of the causative factors for persistent periapical disease [1, 4]. The ability to remove root filling materials is oftentimes most difficult due to anatomical constraints that may prevent thorough cleaning. 

 While a wide range of anatomical complexities may be encountered during root canal retreatment/revision procedures, including fins, webs, cul-de-sacs, isthmuses, ribbon- and dumbbell-shaped canals, dilacerations, and C- and S-shaped canals, the most commonly encountered anatomical challenge may be the curved canal. Furthermore, clinicians often forget that even though the roots may appear straight on a radiograph, curvatures in the third dimension are quite common [5].

The main concern with the removal of filling materials from curved root canals is using instruments with shape memory (Nickel Titanium—NiTi) without altering the integrity of the root canal walls in an adverse way. This is of special importance knowing that canal curvatures are widely variable in all dimensions [6, 7]. Canal curvatures exceeding 30° lead to complications in root canal preparation and cases are considered more complex. Morphology of curved root canals is of great importance in determining the outcome of instrument application, and, in cases of retreatment, inability to remove material adequately from the root canal can invite repeated failures [4].

Specific rotary NiTi file designs for root filling material removal are the ProTaper Universal Rotary Retreatment System (PTUS, Dentsply Maillefer, Ballaigues, Switzerland) and the R-Endo (Micro-Mega, Besançon, France). The manufacturers claim that these systems, in addition to shaping and finishing the root canal, are also effective in the removal of the root filling material from root canals. Both systems have been evaluated as to their ability to remove the previous root filling materials and retained debris from canals systems, with neither system demonstrating 100% effectiveness [3, 8–11].

From a general perspective, most rotary instrument techniques that are designed specifically for the removal of root filling materials will benefit somehow from the use of heat, which they can generate during applications, or solvents that are added to the canal for ease of initial penetration, although variations in outcomes have been noted and the outcomes are inconclusive [12–14]. While one study showed that NiTi rotary instruments removed more gutta-percha when used with a solvent [15], another study found no difference when the same technique was used to remove gutta-percha with and without chloroform [16]. During the use of heat or solvents, an amorphous melt or mixture of the filling material is produced that can be pushed even further into unclean canal irregularities or into the dentinal tubules. This may then require a greater removal of dentin to remove filling materials from within the tubules and enhance the cleanliness of the canal walls [14, 17]. This may result in weakened root canal walls in the apical third of the canal [18]. Regardless of the technique used, thorough debridement has not shown to be achieved [19, 20]. Here again, operator influence on the outcomes has not been considered when performing these technical evaluations, especially when studied in curved canals.

While studies have been performed that address the removal of gutta-percha and zinc-oxide-eugenol and resin sealers using newer NiTi rotary systems, these were done primarily on teeth with straight roots [8, 14–16, 21–23]. Though few studies have addressed the removal of root filling material from curved canals [24, 25], the ability of instruments to remove resin sealers has not been evaluated thus far. This is clinically important considering the fact that epoxy resin sealers are the most commonly used in contemporary endodontics and that they set to a hard mass.

 It was the aim of this study to evaluate the cleanliness of root canal walls after retreatment using two root canal files or systems specifically designed for retreatment and compare the outcomes to the use of Hedström files in severely curved single-rooted human teeth, obturated with gutta-percha and an epoxy resin or zinc-oxide-eugenol sealer. The null hypotheses in this study were as follows: (a) there is no difference between the file systems in removal of the root filling materials and (b) there is no influence of the type of sealer in terms of susceptibility to be removed by the three file systems.

## 2. Materials and Methods

Human single-rooted mandibular first premolars (*n* = 90) were collected and thoroughly cleaned by removing the hard deposits using curettes and the soft deposits by soaking in 5.25% NaOCl for 5 minutes. The specimens were scanned by a cone-beam computed tomography (CBCT) scanner (3D Accuitomo, J. Morita Corporation, Osaka, Japan), and only teeth with root curvatures between 40 and 45 degrees radius of curvature <10 mm were included [26]. The teeth were decoronated at the cementoenamel junction using a diamond disc, under water cooling. The root lengths were standardised to 15 mm. Working length was established using size 10 K-file (Mani Inc, Tochigi, Japan) to the root canal terminus and subtracting 0.5 mm from this measurement. All procedures were performed by a single trained and calibrated operator who demonstrated the same level of proficiency in all the techniques of instrument application for retreatment purposes.

 The root canals were instrumented using nickel titanium rotary instruments (Mtwo, VDW GmbH, Munich, Germany) up to size number 25, 0.07 taper. Irrigation was performed with 3% sodium hypochlorite, using a 5 mL disposable plastic syringe with a polypropylene capillary tip (Ultradent Products Inc., South Jordan, Utah, USA). The tip was placed passively into the canal, up to 2 mm from the apical foramen without binding. All root canals were irrigated with 5 mL of 17% EDTA (Pulpdent, Mass, USA) for 1 minute to remove the smear layer and then rinsed with 5 mL of distilled water. The roots were randomly divided into two groups (*n* = 45) with the aid of a computer algorithm (http://www.random.org/), based on the material used for obturation: Group 1, obturated with gutta-percha and a zinc-oxide-eugenol sealer (Pulp Canal sealer, Sybron Endo, Calif, USA); Group 2, obturated with gutta-percha and an epoxy resin sealer (AH Plus, Dentsply DeTrey, GmbH, Germany). 

The root canals were dried with sterile paper points and obturated with gutta-percha and sealer using continuous wave of warm gutta-percha technique. A System B compactor 0.04 taper tip size 30 (Analytic Technology, Redmond, Wash, USA) was inserted 3-mm short of the working length, with the unit set at 200°C and power 10. After searing off the points at the canal orifices, the activated compactor was pushed apically into the gutta-percha until just short of the premeasured length. The compactor was seated to length without heat and apical pressure was maintained for approximately 10 s. A second burst of heat was used to remove the compacting instrument. Backfilling was performed by injecting thermoplasticized gutta-percha (Obtura II, Obtura Corp, Fenton, Mo, USA). The teeth were radiographed (DSX 730, Owandy Dental Imaging, Champs sur Marne, France) at different angulations to verify the quality of filling procedure. The obturated roots were stored in 100% humidity at 37°C for 1 month.

Following 1 month of storage, the specimens of each group were randomly subdivided into three subgroups (*n* = 15) based on the technique of retreatment. Specimens of subgroup A were retreated using a combination of Gates Glidden drills numbers 3 and 4 (Mani Inc, Tochigi, Japan) in the coronal and middle thirds up to 7 mm in depth, and the remaining portion of the canal was cleaned with H files (Mani Inc, Tochigi, Japan) in a crown-down fashion up to an apical size of 30. The debris was rinsed out with 1% sodium hypochlorite. Specimens of Subgroup B were retreated with the ProTaper retreatment system. The canals were instrumented in a crown-down sequence using ProTaper D1 and D2 files to remove the root filling material. The debris was rinsed from the canal with 1% sodium hypochlorite. 

In Subgroup C, R-Endo instruments (Rm, Re, R1, R2, R3) were used in a gentle in-and-out motion on canal walls according to the manufacturer's instructions. A manual file was used first to relocate the canal orifices, then the Re instrument removed the first 2-3 mm of the filling. R1 and R2 were used on one-third and two-thirds of the estimated working length, respectively. Finally, R3 was used at the working length to complete the removal of filling material from the root canal, and the Rs file was used at full working length to finish the preparation.

All instruments were used only for one specimen, and removal of filling materials was judged complete when the working length was reached, and no more gutta-percha could be seen on the last instrument used. All the teeth were grooved buccolingually with a diamond disc just enough to weaken the tooth to be split into longitudinal sections with a chisel. During the grooving, both the access opening and apical foramen were covered with sticky wax to prevent any debris that may have been generated during the grooving process from getting into the canal and contaminate the root walls. Both halves of the root canal were photographed (Nikon Coolpix 4500; Nikon, Melville, NY, USA) under a stereomicroscope at 40x magnification and analyzed with AutoCAD 2007 software (Autodesk Inc., San Rafael, Calif, USA). The evaluation of coded specimens was performed by 2 operators blinded to the techniques and the devices used for retreatment. 

### 2.1. Data Presentation and Analysis

The arithmetical means of the area of the canal and remaining gutta-percha and sealer (in millimeters), obtained by the 2 operators, were used to measure the percentage of remaining filling materials for all specimens. The intraclass correlation coefficient was calculated to estimate the reliability of the measurements recorded by the 2 examiners. The percentage of remaining filling material and the mean time of gutta-percha removal were evaluated for each group. Descriptive statistics were expressed by means and standard deviations. Data analysis by D'Agostino and Pearson's omnibus normality test showed normal distribution. Consequently, parametric statistical tests were applied. The values were compared statistically by one-way ANOVA with Bonferroni adjustment for multiple comparisons, considering *P* = 0.05 as the level of significance.

## 3. Results

**Figure 1:**
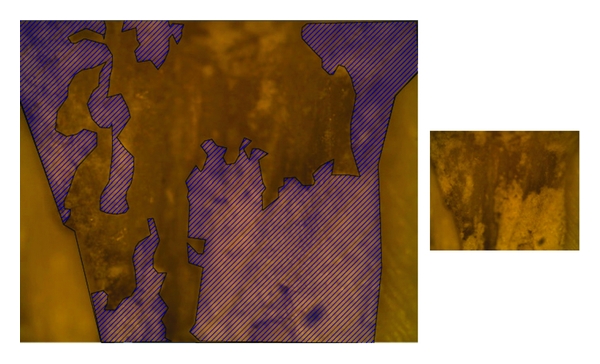
Root filling material remaining in the apical third of a specimen obturated with gutta-percha/AH plus sealer, and retreated using Hedström files.

**Figure 2:**
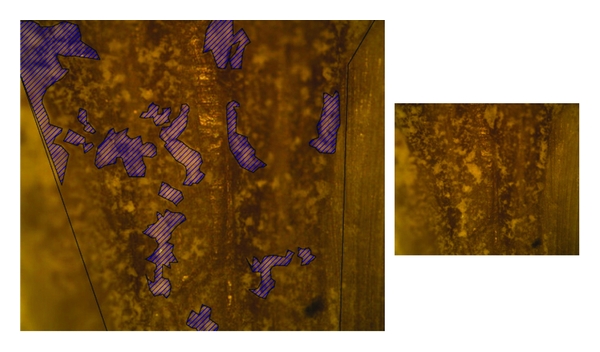
Root filling material remaining in the middle third of a specimen obturated with gutta-percha/ZOE sealer and retreated using R-endo.

The mean percentage of filling material of the retreatment protocols in the different root thirds was tabulated (Table 1), and the value of the intraclass correlation coefficient was very high (*ρ* = 0.99). The apical third of roots obturated with Group II (GP/AH Plus) and retreated with Subgroup A (H files) showed significantly higher (*P* < 0.05) percentage of filling material (65.50 ± 1.17) (Figure 1). The least percentage of filling material was found in the middle third of roots obturated with Group I (GP/ZOE) and retreated with Subgroup C (R-endo) (13.93 ± 0.94) (Figure 2), although this was not significantly different from the coronal and middle third subjected to the same protocols (*P* > 0.05). Analysing the percentage of root filling material considering root thirds as variable, the apical third always had significantly more material than the middle third and coronal third (*P* < 0.05), except in Group I—Subgroups B and C. Disregarding the root third as grouping variable, the R-endo system performed significantly better than the other two file systems (*P* < 0.05).

## 4. Discussion

The present study determined the efficacy of two rotary retreatment systems in comparison with Hedström files to remove gutta-percha obturated in conjunction with an epoxy resin or zinc-oxide-eugenol-based sealer. While studies using NiTi instruments have shown the ability to clean and shape curved root canals with a reasonable degree of safety, thereby preventing or minimizing ledging, transportation, and zipping, their use in the removal of root filling materials and biologic debris has not been investigated extensively [27, 28]. Although newer instruments have been introduced for the specific removal of gutta-percha, sealer, core carriers, and paste fillings, a thorough evaluation of their efficacy in canal debridement and cleaning is lacking in two particular areas: (i) their use in curved canals and (ii) their use relative to the calibration and standardization of the clinician or clinicians who are evaluating the procedures. Only too often studies cite that a specific instrument or technique has failed to achieve an objective, when the application of a specific instrument and the outcome were totally operator dependent. 

Historically and contemporarily, techniques used to remove root canal filling materials have included the use of hand (K-files and Hedström file) or rotary instruments (Gates Glidden burs, Peeso reamers, and NiTi instruments) with or without the use of heat, solvents, and/or ultrasonic applications [10, 29–31]. 

The use of hand files for root canal retreatment has generally been found to be less effective in canal cleaning procedures [10, 32], although some deviations from this finding have been noted with the removal of gutta-percha and synthetic polymer-based materials, such as Epiphany/Resilon (Resilon Research LLC, Madison, Conn, USA) or Endo Rez (Ultradent Products Inc, South Jordan, Utah, USA) [25, 33], and when evaluated using a dental operating microscope [34]. Before the advent of NiTi rotary instruments and the development of specific NiTi instruments for root canal retreatment, the use of Gates Glidden burs and Peeso reamers was commonplace in the removal of gutta-percha, sealers, and pastes. Even recent studies have focused on this approach with variable outcomes being reported [31, 35]. 

The PTUS has three files, each of different lengths, tapers, and apical tip diameters. The instruments—D1 (length = 16 mm, tip diameter = 0.30 mm, 9% taper), D2 (length =18 mm, tip diameter = 0.25 mm, 8% taper), and D3 (length = 22 mm, tip diameter *r* = 0.20 mm, 7% taper) serve to remove filling material from the coronal, middle, and apical root thirds, respectively. The D1 instrument serves to flare the canal walls and has an active tip to facilitate initial penetration into the filling material. The manufacturer recommends that D1 and D2 be used in the coronal and middle thirds, respectively, while D3 should be used to full working length. In the present study, D2 was used up to full working length because the canal was initially prepared to an apical size of 25 with Mtwo instruments. The R-endo files are a system of four files Re (size 25, 0.12 taper) which serves to flare the first few millimetres of the canal, while the other three files R1, R2, and R3 (size 25 with 0.08,0.06, and 0.04 tapers, resp.) are dedicated to the coronal, middle, and apical root thirds, respectively. 

The results of the present study support the results of previous studies in that no retreatment instrumentation protocol is able to completely remove the root filling material [8–10, 13, 16, 21]. Though the ProTaper Universal system was more effective than Hedström files in removal of root filling material, it was less effective than the R-endo system. While the cross-sectional design of the PTUS files favors removal of large amounts of gutta-percha in spirals around the instruments, the same cross-sectional design and the high-centering ability prevent it from contacting all the walls of the root canals, thereby deterring complete removal of filling material from the root canals [10, 22, 36]. The R-endo files, on the other hand, have a triangular cross-section with three equally spaced cutting edges and no radial land.

These results are in contrast to earlier reports where R-endo was compared with PTUS or H files. However, this difference in results may be attributed to the use of solvents in those reports. Solvents may soften the root filling material and thereby compact the material into the irregularities along the root canal wall and dentinal tubules, after which removal may not be possible. Furthermore, solvents may not be considered a standard practice in contemporary endodontics [14]. 

The method used to evaluate the filling remnants plays an important role on the results obtained in each study. Since radiographs are limited to two dimensions, longitudinal cleavage of roots was performed to observe residual remnants on the root canal walls. In order to measure the remnants, commercial software was used to calculate the dentinal wall surface and sealer and gutta-percha remnants. This method has also been used in previous studies [10, 22].

In general all systems were able to remove gutta-percha obturated with zinc-oxide-eugenol more than gutta-percha with resin sealer. Furthermore, the presence of gutta-percha remnants was higher in the apical third of the root for all three groups. This finding suggests the necessity to increase the size of the apical preparation when rotary instruments are used [23]. However, the remaining gutta-percha in the apical third was significantly less in the R-endo group than PTUS and H-files. This may be because of the increased tip diameter of the Rs file (0.30 mm) as compared to the D2 instrument (0.25 mm). 

Furthermore, there is evidence to show that PTUS and H files may cause damage to the root canal dentin during retreatment [18] and that the debris extruded by PTUS during retreatment is less than that by H files [9]. Future studies should focus on comparing the effects of different rotary retreatment systems on damage to root dentin and debris extrusion when used in severely curved canals.

## 5. Conclusions

This in vitro study showed that none of the systems were able to completely remove filling material from the root canals. More specifically,

- application of the R-endo system resulted in less percentage of root filling material on the walls when compared to the ProTaper retreatment system and Hedström files;
- the residual root filling remnants were greater when samples where obturated with an epoxy resin sealer;
- the apical third always had more filling remnants compared to the coronal and middle thirds.

## Figures and Tables

**Table 1:** Percentage (Mean ± S.D) of remaining root filling material in each group at different root-thirds.

GROUP	CORONAL	MIDDLE	APICAL
I (Gutta-percha/ZOE)			
Subgroup A	22.34 ± 0.72^A,*α*^	33.9 ± 0.77^A,*β*^	53.92 ± 0.98^A,*χ*^
Subgroup B	19.21 ± 1.1^B, *α*^	18.62 ± 0.93^B,*α*^	22.81 ± 2.68^B,*α*^
Subgroup C	12.71 ± 1.5^C,*α*^	13.93 ± 0.94^C,*α*^	14.52 ± 0.92^C,*α*^

II (Gutta-percha/AH Plus)			
Subgroup A	42.57 ± 1.1^D,*α*^	59.06 ± 0.98^D,*β*^	65.50 ± 1.17^D,*χ*^
Subgroup B	30.88 ± 1.02^E,*α*^	24.40 ± 0.92^E,*β*^	37.8 ± 1.29^E,*χ*^
Subgroup C	21.80 ± 1.21^A,*α*^	20.21 ± 1.3^F,*α*^	18.58 ± 0.98^B,*β*^

Mean values that share a superscript letter were not significantly different at the 5% level within the same root third (ANOVA, Bonferroni; *P* > 0.05). Between the root-thirds, groups that share the same superscript symbol were not significantly different from each other (*P* > 0.05).
